# Protective Role of Dioscin against Doxorubicin-Induced Chronic Cardiotoxicity: Insights from Nrf2-GPX4 Axis-Mediated Cardiac Ferroptosis

**DOI:** 10.3390/biom14040422

**Published:** 2024-03-30

**Authors:** Jia Liu, Honglin Liu, Liangyan Deng, Tao Wang, Luyao Li, Yuanyuan Chen, Liping Qu, Wenjun Zou

**Affiliations:** State Key Laboratory of Southwestern Chinese Medicine Resources, Chengdu University of Traditional Chinese Medicine, Chengdu 611137, China; liujiafree@stu.cdutcm.edu.cn (J.L.); liuhonglin@stu.cdutcm.edu.cn (H.L.); dengliangyannice@163.com (L.D.); a15037321109@163.com (T.W.); liluyao@stu.cdutcm.edu.cn (L.L.); yuanyuanchen@stu.cdutcm.edu.cn (Y.C.)

**Keywords:** dioscin, doxorubicin, chronic cardiotoxicity, ferroptosis, Nrf2/GPX4 signaling pathway

## Abstract

Recent evidence suggests that ferroptosis, an iron-facilitated cell death with excessive lipid peroxidation, is a critical mechanism underlying doxorubicin (DOX)-induced cardiotoxicity (DIC). Although dioscin has been reported to improve acute DIC, direct evidence is lacking to clarify the role of dioscin in chronic DIC and its potential mechanism in cardiac ferroptosis. In this study, we used chronic DIC rat models and H9c2 cells to investigate the potential of dioscin to mitigate DIC by inhibiting ferroptosis. Our results suggest that dioscin significantly improves chronic DIC-induced cardiac dysfunction. Meanwhile, it significantly inhibited DOX-induced ferroptosis by reducing Fe^2+^ and lipid peroxidation accumulation, maintaining mitochondrial integrity, increasing glutathione peroxidase 4 (GPX4) expression, and decreasing acyl-CoA synthetase long-chain family 4 (ACSL4) expression. Through transcriptomic analysis and subsequent validation, we found that the anti-ferroptotic effects of dioscin are achieved by regulating the nuclear factor-erythroid 2-related factor 2 (Nrf2)/GPX4 axis and Nrf2 downstream iron metabolism genes. Dioscin further downregulates nicotinamide adenine dinucleotide phosphate oxidase 4 (NOX4) and upregulates expression of frataxin (FXN) and ATP-binding cassette B8 (ABCB8) to limit mitochondrial Fe^2+^ and lipid peroxide accumulation. However, Nrf2 inhibition diminishes the anti-ferroptotic effects of dioscin, leading to decreased GPX4 expression and increased lipid peroxidation. This study is a compelling demonstration that dioscin can effectively reduce DIC by inhibiting ferroptosis, which is dependent on the Nrf2/GPX4 pathway modulation.

## 1. Introduction

Doxorubicin (DOX) is a potent topoisomerase II inhibitor with anticancer properties against both hematologic malignancies (such as leukemia and lymphoma) and solid tumors (such as breast, gastric, and osteogenic bone tumors) [1]. Unfortunately, the cardiotoxicity of DOX presents an urgent clinical challenge. Cumulative doses of DOX greater than 400 mg/m^2^ may lead to impaired left ventricular ejection dysfunction, hypertrophic cardiomyopathy, and heart failure [2,3]. Despite breakthrough studies on doxorubicin-induced cardiotoxicity (DIC), including those on multiple overlapping or intersecting cell death pathways, the specific pathogenesis remains unclear [4]. Research has shown that DOX accumulation in mitochondria is 100 times greater than in plasma, making myocardial mitochondria key organelles in DIC [5]. Ferroptosis is manifested by fatal iron overload and aggregation of lipid peroxides [6]. A report in 2019 demonstrated the presence of ferroptosis in DIC, with excess iron in mitochondria leading to lipid peroxidation damage [7]. In 2020, Tadokoro et al. discovered that DOX forms a DOX-Fe^2+^ complex in mitochondria, generating excessive lipid peroxidation and leading to mitochondria-dependent ferroptosis [8]. Recent studies have indicated that DOX is taken up by mitochondria through integration into mitochondrial DNA, disrupting heme synthesis in cardiomyocytes and inducing mitochondrial iron accumulation, which leads to ferroptosis [9]. These reports suggest that DOX and iron accumulation in mitochondria synergistically induce cardiac ferroptosis. Therefore, targeted intervention of ferroptosis may be a potential method to ameliorate DIC.

Nrf2 regulates many processes associated with ferroptosis, including heme synthesis, iron storage and release, the mercaptan-dependent antioxidant system, and reactive oxygen species (ROS) production both inside and outside the mitochondria [10]. Research has reported that DOX treatment inhibits Nrf2 expression, leading to an imbalance in downstream iron metabolism genes such as divalent metal transporter (DMT1), ferritin light chain/heavy chain (FTL/FTH1), and ferroportin (FPN), ultimately resulting in iron aggregation and disruption of the antioxidant system, triggering a fatal lipid peroxidation reaction [11,12]. GPX4, the unique member of the glutathione peroxidase family that protects biofilms from peroxidation, is a downstream target of the oxidation regulator Nrf2 [13]. As a central regulator of ferroptosis, the GPX4-dependent antioxidant capacity was significantly reduced in the DIC model [14,15]. Combined with previous studies, Nrf2/GPX4 axis activation reduces ROS and lipid peroxidation levels, and also negatively regulates ferroptosis through maintaining iron homeostasis and mitochondrial function [16,17]. Therefore, regulation of the Nrf2/GPX4 pathway is an important research direction to inhibit ferroptosis in DIC.

Dioscin is a spiro-stanol saponin generally found in edible vegetables and herbs, mostly belonging to the family of Dioscoreaceae. Dioscin has attracted attention for its antitumor, anti-inflammatory, and cardioprotective effects [18]. Regarding its cardioprotective properties, the antioxidant, anti-apoptotic, and anti-inflammatory properties of dioscin have been shown to be effective in alleviating diabetic cardiomyopathy, heart failure, and DIC [19]. Specifically, in DOX-induced acute rat/mouse models (a single intraperitoneal injection of 15 mg/kg DOX for 7 days), dioscin has been observed to protect against DIC by reducing pathological damage and ameliorating cardiac dysfunction. This protective mechanism is associated with the modulation of miR-140-5p-mediated myocardial oxidative stress under the initiation of the Nrf2/sirtuin 2 pathway [20]. Additionally, Yuan et al. reported that dioscin modulates the phosphoinositide-dependent protein kinase-1 (PDK1)-regulated Akt/mammalian target of rapamycin (mTOR) pathway to improve DOX-induced heart failure (intraperitoneal injection of 2.5 mg/kg DOX six times in 2 weeks) [21]. These results provide evidence that the therapeutic effect of dioscin on acute DIC may be related to Nrf2-associated oxidative stress. However, recent reports have placed more emphasis on the importance of Nrf2 activation in inhibiting ferroptosis in DIC [22,23]. Although the cardioprotective effect of dioscin in acute DIC has been confirmed, its effect on cumulative cardiotoxicity of DOX is more worthy of investigation. This study was the first to investigate whether dioscin can attenuate chronic DIC by inhibiting ferroptosis through Nrf2/GPX4 pathway activation. We present evidence from both H9c2 cells and chronic DIC rat models that delineate the pathways by which dioscin exerts its effects, with a particular focus on its interaction with the Nrf2/GPX4 axis.

## 2. Materials and Methods

### 2.1. Reagents and Materials

DOX (S1208), Fer-1 (S7243), erastin (S7242), and ML385 (S8790) were purchased from Selleck (Shanghai, China). Dioscin (E-0098, purity: 98%) was obtained from Shanghai Tauto Biochemical Technology Co., Ltd. (Shanghai, China). Cell culture material including Dulbecco’s modified Eagle’s medium (DMEM, 11965-092), fetal bovine serum (FBS, 10099-141), and antibiotics (penicillin and streptomycin, 15070-063) were purchased from Gibco Co., Ltd. (Grand Island, NY, USA). 4′,6′-Diamidino-2-phenylindole (DAPI, P0131), JC-1 (C2003S) and DCFH-DA (S0033S) assay kits, MitoTracker red CMXRos (C1035), Mito-Tracker Green (C1048), and RIPA lysis buffer (P0013B) were supplied by Beyotime Institute of Biotechnology (Nanjing, China). DyLight 488 Conjugated AffiniPure Goat Anti-rabbit IgG (H+L) (BA1227) and DyLight 550 Conjugated AffiniPure Goat Anti-rabbit IgG (H+L) (BA1135) were purchased from the Boster Biological Technology Company (Wuhan, China). Myocardial enzyme assay kits including CK-MB (105-000459-00), LDH (105-000446-00), AST (105-000443-00), and ALT (105-000442-00) were purchased from Shenzhen Mindray Bio-Medical Electronics Co. (Shenzhen, China). Oxidative stress detection kits including SOD (A001-3-2), CAT (A007-1-1), GSH (A006-2-1), GSH-Px (A005-1-2) and MDA (A003-1-2) were purchased from Nanjing Jiancheng Institute of Biotechnology (Nanjing, China). ELISA kits for cTnT (E-EL-R0151), 4-HNE (E-EL-0128), and total iron colorimetric assay kits (E-BC-K772-M) were obtained from Elabscience (Wuhan, China). FerroOrange (F374), Mito-FerroGreen (M489), and MitoPeDPP (M466) probes were supplied by Dojindo Molecular Technologies (Kumamoto, Japan). C11-BODIPY581/591 (HY-D1301) and MitoSox Red (HY-D1055) were obtained from MedChemExpress (Shanghai, China). Anti-Nrf2 (A21176), anti-ABCB8 (A2653) antibodies, and HRP Goat Anti-Rabbit IgG (H+L) (AS014) were obtained from ABclonal Technology (Wuhan, China). Anti-GPX4 (DF6701), anti-HO-1 (AF5393), anti-NOX4 (DF6924), anti-ACSL4 (DF12141), anti-TfR1 (AF5343), anti-FXN (DF6590), and β-tubulin (AF7011) were purchased from Affinity Biosciences (Liyang, China). Total RNA isolation kit (RE-03014), RT EasyTM II (RT-01022), and Real Time PCR assay kit (QP-01014) were obtained from Foregene Co., Ltd. (Chengdu, China). siRNA was purchased from GenePharma, and siRNA transfection kit (40808ES) was obtained from Yeasen Biotechnology Co., Ltd. (Shanghai, China).

### 2.2. Animal Experimental Design and Model Establishment

Male Sprague-Dawley (SD) rats (8 weeks old, 220–250 g) were obtained from Si Pei Fu Biological Technology Co., Ltd. (Beijing, China). After 7 days of acclimatization, 30 rats were divided into three groups: a control group receiving intraperitoneal administration of 0.9% normal saline with an equal volume in the DOX group, a DOX group receiving intraperitoneal administration of 2.5 mg/kg/week DOX for 6 weeks (DOX was dissolved in 0.9% saline), and a DOX+dioscin treatment group receiving intragastric administration of 60 mg/kg/day dioscin (dioscin was dissolved in 0.5% CMC-Na) and intraperitoneal administration of 2.5 mg/kg/week DOX for 6 weeks. The dosage of dioscin was determined based on a previous DOX cardiotoxicity study [20]. Prior to sacrifice, rats were evaluated by electrocardiogram and echocardiogram. Blood was centrifuged for 10 min to obtain serum, and cardiac tissues were collected for subsequent pathological staining and analysis of relevant proteins and genes. The experiment was approved by the Animal Experiment Ethics Committee of Chengdu University of Traditional Chinese Medicine (Approval number: SYXK 2020-124; date of approval, 30 June 2020).

### 2.3. Measurement of Electrocardiograms and Echocardiography

After removal of chest hair, all rats were anesthetized with oxygen containing 1–1.5% isoflurane. The BL-420F Biological Function Experimental System (Chengdu Tailian Technology Co., Ltd., Chengdu, China) was connected to the animals using hypodermic needle electrodes, and stable electrocardiogram data were recorded. In addition, the Vevo 3100 system with a 40 MHz probe (FUJIFILM visualsonic, Inc., Toronto, ON, Canada) was utilized to assess cardiac function parameters.

### 2.4. Determination of Cardiac Injury Markers and Iron Content

Myocardial enzymes including CK-MB, LDH, AST, and ALT levels were measured using the BS-360S automatic serum biochemical analyzer (Mindray, Shenzhen, China) [24]. The heart tissue was added to a homogenizer machine with a corresponding volume of cold PBS (1:9, *w*/*v*). The homogenized supernatant was used to determine the levels of cTnT and 4-HNE in cardiac tissues according to the ELISA kit. For SOD, CAT, GSH, and MDA levels in cardiac tissues, 10% homogenized supernatant was collected for content assay according to the instructions. Additionally, 0.1 g of fresh heart tissues were homogenized with 0.9 mL of extraction solution and centrifuged at 12,000× *g* for 10 min to obtain a supernatant for the determination of cardiac tissue iron.

### 2.5. Histopathologic Examination and Prussian Blue Staining

Cardiac tissues were fixed, dehydrated, embedded in wax and sectioned. Pathological changes in the cardiac tissue were detected using hematoxylin-eosin staining. Images of the stained sections were captured using a Nikon microscope (Nikon Corporation, Tokyo, Japan). For Prussian blue staining, the slides were immersed in 1% potassium ferricyanide for one hour. Images of this staining were also captured with a Nikon microscope.

### 2.6. Cell Culture and Viability Assay

The H9c2 cell line (catalog no. GNR 5) was obtained from the Cell Bank of the Chinese Academy of Sciences (Shanghai, China). The cells were cultured in DMEM growth medium containing 10% FBS and 1% penicillin-streptomycin at 37 °C with 5% CO_2_. Dioscin, DOX, Fer-1, and erastin were dissolved in DMSO (final concentration of DMSO was less than 0.1%) and diluted with DMEM (2% FBS) to final concentrations. H9c2 cells were cultured in 96-well plates at a density of 5 × 10^4^ cells/mL for 24 h. Then, H9c2 cells were treated with different concentrations of dioscin (0–8 μM), DOX (0–10 μM), Fer-1 (0–20 μM), and erastin (0–8 μM) for 24 h to observe cytotoxicity. To observe the protective concentration of dioscin, H9c2 cells were pretreated with different concentrations of dioscin (0–2000 nM) for 24 h and then treated with DOX (4 μM) for 24 h. To observe the protective concentration of Fer-1, H9c2 cells were co-treated with different concentrations of Fer-1 (0–8 μM) and DOX (4 μM) for 24 h. In addition, H9c2 cells were pretreated with dioscin (0–2000 nM) for 24 h followed by induction with erastin (1 μM) for 24 h to assess its inhibitory effect on ferroptosis. The cells in control groups were treated with 0.1% DMSO. A CCK-8 solution (10 μL) was added to the plates and incubated until it turned orange. The absorbance was then measured at 450 nm using a Bio-Rad multiple detection microplate reader (Bio-Rad, San Diego, CA, USA).

### 2.7. Transmission Electron Microscopy

Fresh cardiac tissue (1 m^3^) was fixed with electron microscope fixative solution for 2–4 h. H9c2 (1 × 10^6^ cells/dish) were inoculated onto 6 cm dishes and incubated with dioscin (200 nM) for 24 h before DOX (4 μM) treatment. Cells were collected by trypsin digestion, supernatants were discarded, and fixed with electron microscope fixative solution for 2–4 h. Cardiac tissue and cell samples were then fixed with 1% osmic acid for 2 h, and then washed with PBS. The specimens underwent ethanolic dehydration, epoxy embedding, and ultramicrotome sectioning. The samples were processed with uranyl acetate and lead citrate, and images were captured using transmission electron microscopy (JEOL, Tokyo, Japan).

### 2.8. Cellular ROS, Oxidative Stress Parameters, Fe^2+^ and Lipid ROS Assay

H9c2 cells were grown in 6-well plates at a density of 1 × 10^6^ cells/mL and cultured with dioscin (100, 200, and 400 nM) for 24 h prior to DOX (4 μM)-induced cytotoxicity. After replacing the old medium, the cells were incubated with DCFH-DA (10 μM, 1 mL) for 30 min at 37 °C. The cells were then washed three times with PBS, collected, and analyzed using FACSCanto II flow cytometry (BD, New York, NY, USA). In addition, cells from 6-well plates were digested with trypsin after drug treatment, and the precipitated cells were broken by sonication. The supernatant was collected and assayed for SOD, CAT, GSH, GSH-Px, and MDA levels according to the kit instructions. Similarly, the cell-broken supernatant was collected and assayed for 4-HNE levels according to the ELISA kit. H9c2 cells (1 × 10^5^ cells/mL) were seeded into confocal laser dishes and cultured with dioscin (200 nM) and DOX (4 μM). The medium was then removed and the cells were washed three times with HBSS. H9c2 cells were then stained with 1 μM FerroOrange for 30 min to observe intracellular Fe^2+^ levels. Similarly, H9c2 cells were stained with 2 μM C11-BODIPY581/591 probes for 30 min to observe lipid ROS levels. Cell fluorescence intensity was observed using a confocal fluorescence microscope (Leica Microsystems, Wetzlar, Germany).

### 2.9. Mitochondrial Membrane Potential, Mitochondrial Fe^2+^, ROS and Lipid ROS Assay

JC-1 working solution (1 mL) was added to each confocal dish, and the cells were incubated for 20 min before observation under a laser confocal microscope. To detect intracellular mitochondrial ROS levels, the cells were first incubated with MitoTracker Green (200 nM) for 30 min to localize mitochondria. The cells were then incubated with MitoSox Red (5 μM) for 10 min. For Mito-FerroGreen staining, cells were incubated with MitoTracker red CMXRos (200 nM) for 15 min to localize mitochondria. H9c2 cells were then cultured with Mito-FerroGreen (5 μM) for 30 min. For MitoPeDPP staining, the cells were cultured with MitoPeDPP working solution (0.5 μM) for 15 min. Photographs were then captured using confocal microscopy, and fluorescence intensity was analyzed.

### 2.10. Immunofluorescence Staining

H9c2 cells (1 × 10^5^ cells/mL) were seeded into confocal laser dishes. Following dioscin (200 nM) pretreatment for 24 h and DOX (4 μM) intervention for 24 h, H9c2 cells were fixed with fresh 4% paraformaldehyde for 20 min at room temperature. Cells were then permeabilized with 0.2% Triton X-100 for 15 min and blocked with 5% goat serum for 30 min. Antibodies against Nrf2 (A21176, ABclonal), NOX4 (DF6924, Affinity), and GPX4 (DF6701, Affinity) (antibody dilution ratio 1: 200) were incubated overnight at 4 °C, respectively. The cells were then incubated with the DyLight 488 Conjugated AffiniPure Goat Anti-rabbit IgG (H+L) (1:200, BA1227, Boster) at room temperature for one hour. Similarly, cardiac tissue sections were permeabilized with a blocking solution containing 0.2% Triton X-100 and 5% goat serum for 30 min. Then, cardiac tissue sections were treated overnight with Nrf2 (A21176, ABclonal), NOX4 (DF6924, Affinity), and GPX4 (DF6701, Affinity) antibodies (antibody dilution ratio 1: 100), respectively, followed by incubation with DyLight 550 Conjugated AffiniPure Goat Anti-rabbit IgG (H+L) (1:200, BA1135, Boster). Fluorescence changes were captured using confocal laser microscopy and quantified using ImageJ software version 2.0.

### 2.11. Transcriptome Sequencing and KEGG and PPI Analysis

H9c2 cells were treated with DOX (4 μM), with or without dioscin (200 nM). Total RNA was isolated by the TRIzol method and reverse-transcribed to cDNA. High-throughput sequencing was performed using the Illumina HiSeq after quality assessment. Differentially expressed genes were identified with a threshold of |log2FC| > 1 and *p*-adjust < 0.05. Enrichment analyses were then conducted to elucidate the biological significance of DOX or dioscin on these genes.

### 2.12. Real-Time Quantitative PCR (RT-qPCR)

Total RNA was extracted from cardiac tissue or H9c2 cells using an RNA isolation kit. RNA purity was assessed using a nucleic acid/protein analyzer with OD260/280 values. Reverse transcription was carried out according to the reverse transcription reaction conditions using 2 × RT OR-EasyTM Mix to produce stabilized cDNA. RT-qPCR was performed on the stabilized cDNA and specific primers according to the manufacturer’s instructions. GAPDH was used as an endogenous control mRNA, and the data were analyzed using the 2^−ΔΔCT^. The sequences of the primers are listed in Table 1.

### 2.13. Western Blotting Assay

Total protein samples from cardiac tissue or H9c2 cells were obtained with RIPA lysis buffer (strong) containing 1% protease inhibitors and protein concentrations were determined. After concentration determination, proteins were separated by SDS-PAGE (7.5–12.5%) and transferred to PVDF membranes. The membranes were blocked with 5% skim milk and incubated overnight at 4 °C with primary antibodies Nrf2 (A21176, ABclonal), HO-1 (AF5393, Affinity), TfR1 (AF5343, Affinity), GPX4 (DF6701, Affinity), NOX4 (DF6924, Affinity), ABCB8 (A2653, ABclonal), FXN (DF6590, Affinity) (antibody dilution ratio 1: 1000) and β-tubulin (1:3000, AF7011, Affinity), respectively. The membranes were then incubated with the secondary antibody HRP Goat Anti-Rabbit IgG (H+L) (1:5000, AS014, ABclonal) for 2 h at room temperature. Protein bands detected by an enhanced chemiluminescence system were analyzed using ImageJ software.

### 2.14. Inhibition of Nrf2 in H9c2 Cells

H9c2 cells were cultured in 6-well plates at a density of 2 × 10^4^ cells/well. The next day, Nrf2 siRNA (sense primer 5′-GUAGUCCACAUUUCCUUCATT-3′ and antisense primer 5′-UGAAGGAAAUGUGGACUACTT-3′) and si-control were transfected into H9c2 cells using Lipofectamine 3000. The medium was changed to culture medium after 6 h. After 24 h of transfection, cells were pretreated with dioscin (200 nM) for 24 h, followed by DOX (4 μM) for 24 h. H9c2 cells were harvested for protein analysis. Additionally, we also used the Nrf2-specific inhibitor ML385 to further observe the inhibition of Nrf2 in H9c2 cells. The cytotoxicity of ML385 was evaluated by CCK-8 and the inhibitory concentration of ML385 in H9c2 was determined by RT-qPCR. H9c2 cells were seeded in 6-well plates and pretreated by dioscin (200 nM) with or without ML385 (1 μM) for 24 h [25] before DOX (4 μM) treatment. H9c2 cells were collected for protein analysis. Similarly, H9c2 cells were inoculated onto confocal laser dishes to observe the changes in lipid ROS levels after cell transfection or ML385 treatment.

### 2.15. Data Analysis

Statistical analysis was conducted using GraphPad Prism 8.0 software. One-way analysis of variance (ANOVA) and non-parametric tests were used to compare multiple groups, and unpaired *t*-tests were used to compare two different groups. *p*-values less than 0.05 were considered statistically significant.

## 3. Results

### 3.1. Dioscin Protects against Cardiac Injury and Dysfunction in Chronic DIC Rats

**Figure 1:**
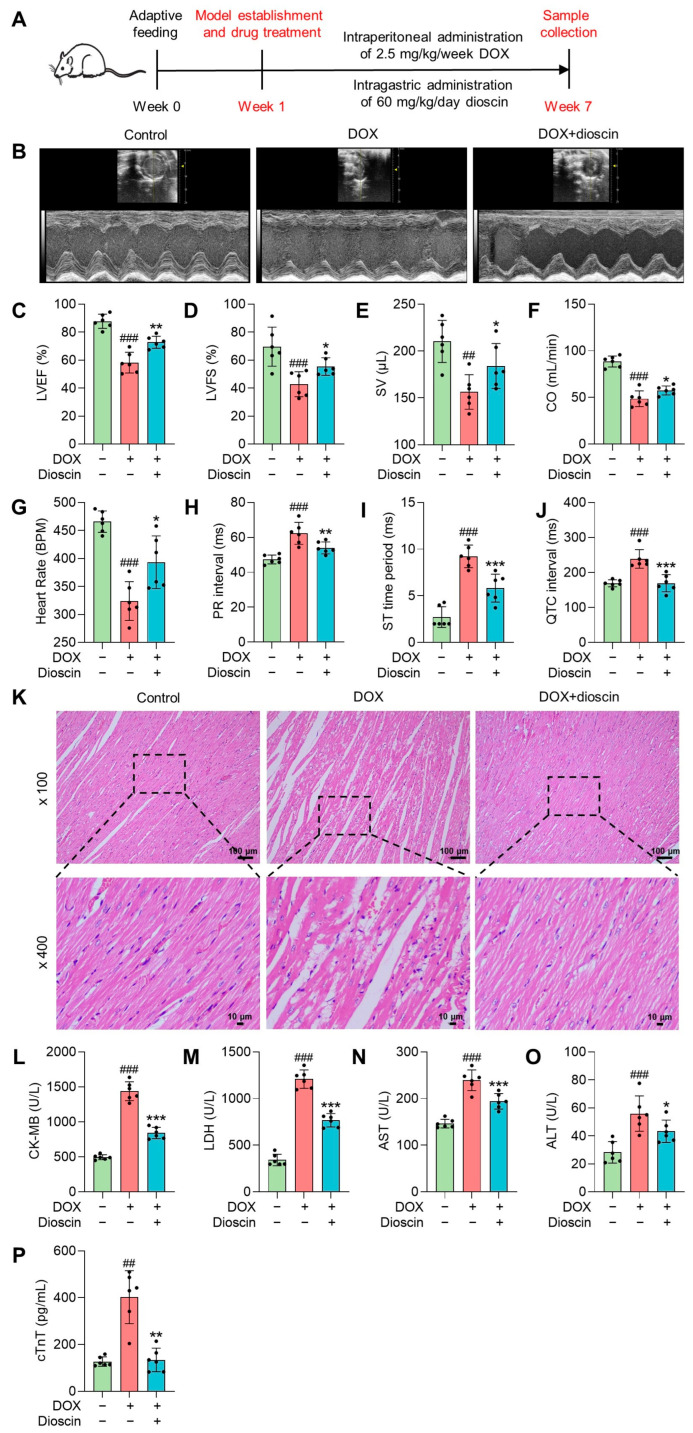
Dioscin exerted protective effects on chronic DIC rats. (**A**) Schematic plans of the animal experiment process. (**B**) Representative echocardiography images of DOX-induced rats treated with or without dioscin. (**C**–**F**) Bar charts indicate the levels of LVEF, LVFS, SV, and CO in DOX-induced rats treated with or without dioscin. Data are mean ± SD (*n* = 6). (**G**–**J**) Bar charts indicate the levels of heart rate, PR interval, ST time period, and QTC interval in DOX-induced rats treated with or without dioscin. Data are mean ± SD (*n* = 6). (**K**) Representative HE-staining images of DOX-induced rats treated with or without dioscin. (**L**–**P**) Bar charts indicate serum levels of CK-MB, LDH, aspartate aminotransferase (AST), alanine transaminase (ALT), and cTnT in DOX-induced rats treated with or without dioscin. Data are mean ± SD (*n* = 6). * *p* < 0.05, ** *p* < 0.01, *** *p* < 0.001 vs. DOX treatment group; ## *p* < 0.01, ### *p* < 0.001 vs. control group.

The animal experimental process is outlined in a schematic diagram shown in Figure 1A. When cardiac function was assessed by echocardiography, significant decreases in left ventricular ejection fraction (LVEF), left ventricular fractional shortening (LVFS), stroke volume (SV), and cardiac output (CO) were observed in the DOX group (Figure 1B–E). Correspondingly, the electrocardiography analysis of the DOX group rats revealed a decreased heart rate, prolonged duration of the PR interphase and QTC interphase, and an increased duration of the ST segment (Figure 1F–I). These findings suggest that DOX-induced myocardial injury is linked to cardiac ferroptosis. Histological analysis showed inflammatory cell infiltration and vacuoles in cardiac tissue after DOX treatment, along with watery lesions in cardiomyocytes (Figure 1J). Serum cardiac injury markers such as creatine kinase isoenzyme-MB (CK-MB), lactate dehydrogenase (LDH), and cardiac troponin T (cTnT) also increased significantly (Figure 1K–O). Treatment with dioscin protected against DOX-induced cardiac dysfunction, not only by reducing levels of cardiac injury markers but also by improving cardiac histopathologic changes. In addition, dioscin administration restored the reduced levels of LVEF, LVFS, SV, and CO, and corrected abnormal electrocardiogram signals. Therefore, dioscin effectively alleviated cardiac injury and dysfunction in chronic DIC rats.

### 3.2. Dioscin Alleviates Cardiac Ferroptosis in DOX-Induced Rats

**Figure 2:**
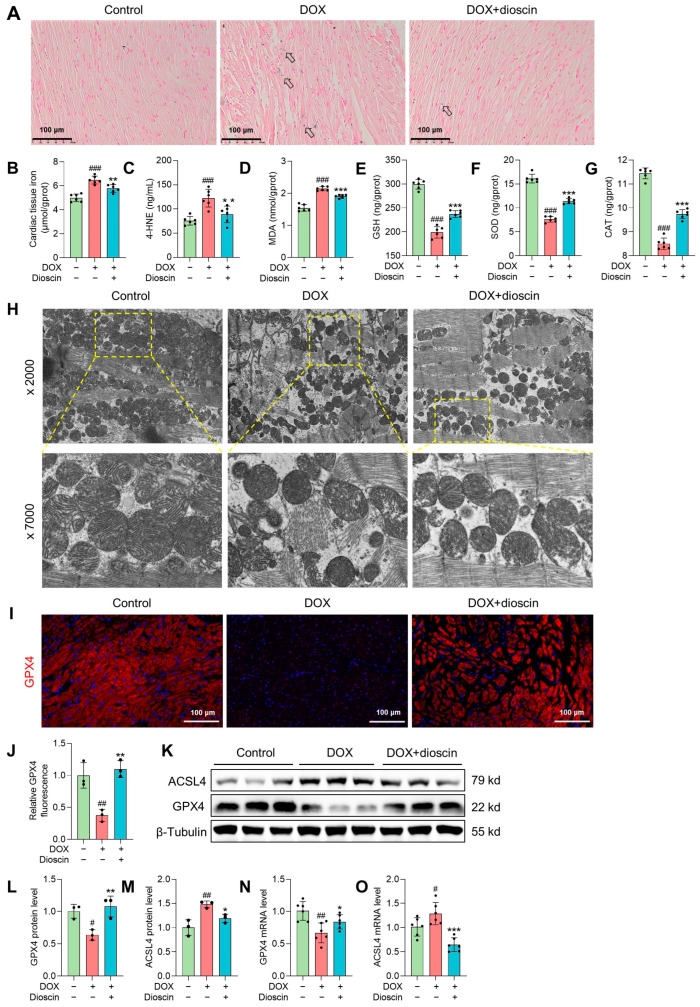
Dioscin alleviates cardiac ferroptosis in DOX-induced rats. (**A**) Representative Prussian blue staining images of DOX-induced rats treated with or without dioscin. The arrows indicate iron deposition in cardiac tissue. (**B**) Bar charts indicate cardiac tissue iron in DOX-induced rats treated with or without dioscin. Data are mean ± SD (*n* = 6). (**C**–**G**) Bar charts indicate serum 4-HNE levels and cardiac tissue MDA, glutathione (GSH), superoxide dismutase (SOD), and catalase (CAT) levels of DOX-induced rats treated with or without dioscin. Data are mean ± SD (*n* = 6). (**H**) The mitochondrial structure of cardiac tissues of DOX-induced rats treated with or without dioscin. (**I**) Representative images of immunofluorescence analysis of GPX4 expression were captured by fluorescence microscopy. (**J**) The bar chart indicates the GPX4 intensity in cardiac tissue. Data are mean ± SD (*n* = 3). (**K**) Representative Western blotting images of GPX4 and ACSL4 in DOX-induced rats treated with or without dioscin. (**L**,**M**) Bar charts indicate the protein expression levels of ACSL4 and GPX4 in cardiac tissues. Data are mean ± SD (*n* = 3). (**N**,**O**) Bar charts indicate the mRNA expression levels of ACSL4 and GPX4 in cardiac tissues. Data are mean ± SD (*n* = 6). * *p* < 0.05, ** *p* < 0.01, *** *p* < 0.001 vs. DOX treatment group; # *p* < 0.05, ## *p* < 0.01, ### *p* < 0.001 vs. control group. (**K**) Original western blotting figures can be found in Figure S3.

In this study, significant iron accumulation in cardiac tissue was observed in DOX-treated rats, consistent with the high iron levels characteristic of ferroptosis (Figure 2A,B). Lipid peroxidation, a key feature of ferroptosis, was evidenced by increased levels of 4-hydroxynonenal (4-HNE) and malondialdehyde (MDA) after DOX administration (Figure 2C,D), along with decreased levels of antioxidant enzymes (Figure 2E–G). Mitochondrial abnormalities, such as disruption of mitochondrial crests, increased porosity, membrane density, and membrane rupture in DOX-treated rats, were observed by electron microscopy (Figure 2H). These findings suggest that DOX-induced cardiac ferroptosis is linked to myocardial injury in chronic DIC. Notably, dioscin treatment attenuated iron overload and mitochondrial damage in DIC rats, reversed the changes in MDA and 4-HNE levels, and restored antioxidant enzyme activity. ACSL4, a key enzyme in the metabolism of polyunsaturated fatty acids (PUFA) and a determinant of ferroptosis susceptibility, was found to be upregulated in DOX-treated rats. In contrast, GPX4, a known inhibitor of ferroptosis, was downregulated [26]. Remarkably, dioscin treatment counteracted the ferroptotic cardiac injury induced by DOX via upregulating GPX4 expression and downregulating ACSL4 expression (Figure 2I–O). These results suggest that dioscin effectively inhibits DOX-produced lipid peroxidation and possesses anti-ferroptotic properties.

### 3.3. Dioscin Alleviates Ferroptosis in DOX-Induced H9c2 Cells

**Figure 3:**
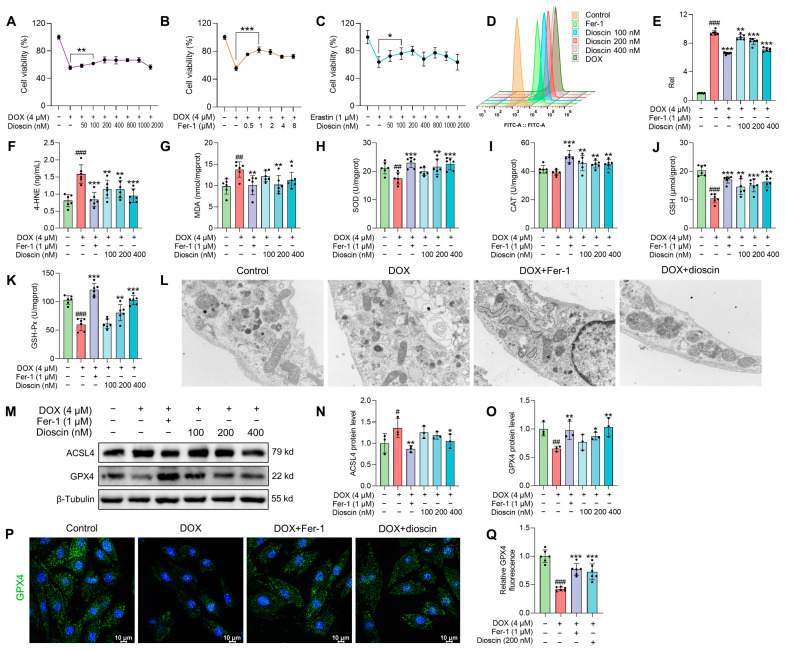
Dioscin alleviates ferroptosis in DOX-induced H9c2 cells. (**A**) The cell viability of H9c2 cells treated with DOX in the presence or absence of dioscin at the indicated concentrations. Data are mean ± SD (*n* = 6). (**B**) The cell viability of H9c2 cells treated with DOX in the presence or absence of Fer-1 at the indicated concentrations. Data are mean ± SD (*n* = 6). (**C**) The cell viability of H9c2 cells treated with erastin in the presence or absence of dioscin at the indicated concentrations. Data are mean ± SD (*n* = 6). (**D**) Representative flow cytometry images showing the DCFH-DA intensity of H9c2 cells treated with DOX in the presence or absence of dioscin and Fer-1. (**E**) The bar chart indicates the ROS production in H9c2 cells. Data are mean ± SD (*n* = 6). (**F**–**K**) H9c2 cells were treated with DOX in the presence or absence of dioscin and Fer-1. Bar charts indicate the levels of 4-HNE, MDA, SOD, CAT, GSH, and GSH-Px. Data are mean ± SD (*n* = 6). (**L**) The mitochondrial structure of DOX-induced H9c2 cells treated with or without dioscin and Fer-1. (**M**) Representative Western blotting images of GPX4 and ACSL4 in DOX-induced H9c2 cells treated with or without dioscin and Fer-1. (**N**,**O**) Bar charts indicate the protein expression levels of ACSL4 and GPX4 in H9c2 cells. Data are mean ± SD (*n* = 3). (**P**) Representative images of immunofluorescence analysis of GPX4 expression were captured by fluorescence microscopy. (**Q**) The bar chart indicates the GPX4 intensity in H9c2 cells. Data are mean ± SD (*n* = 3). * *p* < 0.05, ** *p* < 0.01, *** *p* < 0.001 vs. DOX treatment group; # *p* < 0.05, ## *p* < 0.01, ### *p* < 0.001 vs. control group. (**M**) Original western blotting figures can be found in Figure S4.

The survival rate of H9c2 cells remained unchanged after treatment with dioscin, indicating that dioscin had no significant toxic effect on H9c2 cells (Figure S1A). However, DOX treatment significantly reduced the viability of H9c2 cells, with the survival rate at 4 μM being only 56.58 ± 3.93% (Figure S1B). Ferrostatin-1 (Fer-1), a ferroptosis inhibitor, exhibited noticeable toxicity at 10 μM, where the cell survival rate began to decrease (Figure S1C). Erastin, a ferroptosis inducer, markedly inhibited the proliferation of H9c2 cells at 1 μM and reduced viability by approximately 90% at 2 μM (Figure S1D). Interestingly, dioscin at concentrations of 100, 200, and 400 nM increased the survival rate of H9c2 cells compared to the DOX (4 μM) group (Figure 3A). Fer-1 was most effective in inhibiting DOX cytotoxicity at 1 μΜ, which was chosen as a positive control for subsequent ferroptosis inhibition (Figure 3B). Notably, similar to the ferroptosis inhibitory effect of Fer-1, dioscin (100, 200, and 400 nM) eliminated cell death induced by erastin (1 μM) in H9c2 cells (Figure 3C). In addition, there was a significant increase in ROS accumulation in DOX-induced cells, but dioscin and Fer-1 significantly reduced this DOX-induced ROS production (Figure 3D,E). Dioscin and Fer-1 also decreased MDA and 4-HNE levels and increased antioxidant enzyme activity, as shown in Figure 3F–K. These results suggest that DOX can induce ferroptosis-like cell death, and the ferroptosis-inhibiting properties of dioscin may help protect cardiomyocytes from DOX damage.

**Figure 4:**
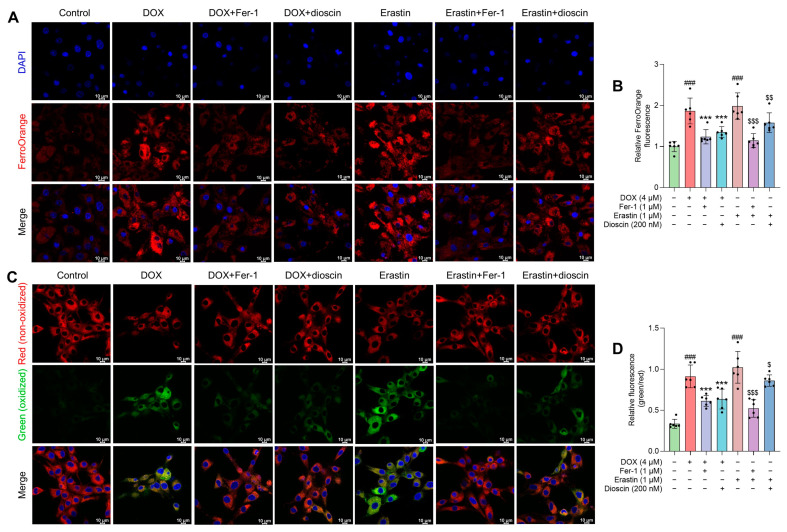
Effects of dioscin on DOX-induced intracellular Fe^2+^ and lipid ROS in H9c2 cells. (**A**) Representative FerroOrange staining images of DOX/erastin-induced H9c2 cells treated with or without dioscin and Fer-1. (**B**) The bar chart indicates the relative levels of Fe^2+^ in H9c2 cells. Data are mean ± SD (*n* = 3). (**C**) Representative C11 BODIPY 581/591 staining images of DOX/erastin-induced H9c2 cells treated with or without dioscin and Fer-1. (**D**) The bar chart indicates the relative levels of lipid ROS in H9c2 cells. Data are mean ± SD (*n* = 3). *** *p* < 0.001 vs. DOX treatment group; ### *p* < 0.001 vs. control group; $ *p* < 0.05, $$ *p* < 0.01, $$$ *p* < 0.001 vs. erastin group.

Characteristic morphological changes in mitochondria associated with ferroptosis were observed. Transmission electron microscopy studies revealed that both Fer-1 and dioscin prevented DOX-induced mitochondrial ridge loss and outer membrane rupture in H9c2 cells (Figure 3L). Consistent with in vivo ferroptosis markers, protein and immunofluorescence analysis demonstrated that dioscin significantly increased GPX4 and decreased ACSL4 expression, exhibiting the same ferroptosis inhibitory effect as Fer-1 (Figure 3M–Q). Additionally, intracellular Fe^2+^ and lipid ROS levels were measured using FerroOrange and C11-BODIPY581/591 probes. When H9c2 cells were treated with DOX or erastin, the intracellular Fe^2+^ and lipid ROS levels increased significantly, but dioscin decreased these ferroptosis-related characteristics (Figure 4A–D). This is consistent with the inhibitory effect of dioscin on cardiac iron levels and lipid peroxides 4-HNE and MDA, suggesting that dioscin can reduce cardiomyocyte ferroptosis.

### 3.4. Dioscin Alleviates DOX-Induced Mitochondrial Ferroptosis in H9c2 Cells

**Figure 5:**
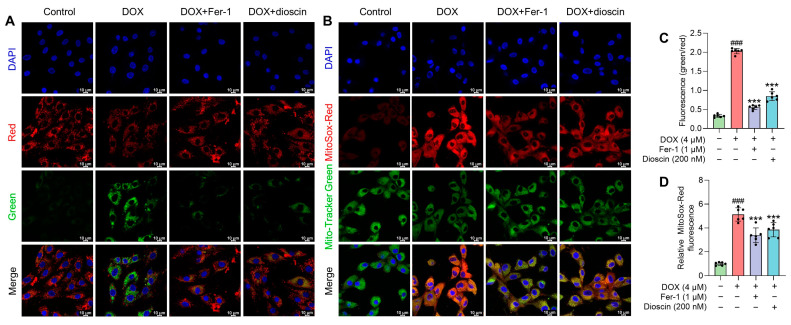
Effect of dioscin on MMP and mitochondrial ROS accumulation during DOX exposure. (**A**) Representative JC-1 staining images of DOX-induced H9c2 cells treated with or without dioscin and Fer-1. (**B**) The bar chart indicates the MMP dissipation in H9c2 cells. Data are mean ± SD (*n* = 3). (**C**) Representative MitoSox Red staining images of DOX-induced H9c2 cells treated with or without dioscin and Fer-1. (**D**) The bar chart indicates the relative levels of mitochondrial ROS in H9c2 cells. Data are mean ± SD (*n* = 3). *** *p* < 0.001 vs. DOX treatment group; ### *p* < 0.001 vs. control group.

**Figure 6:**
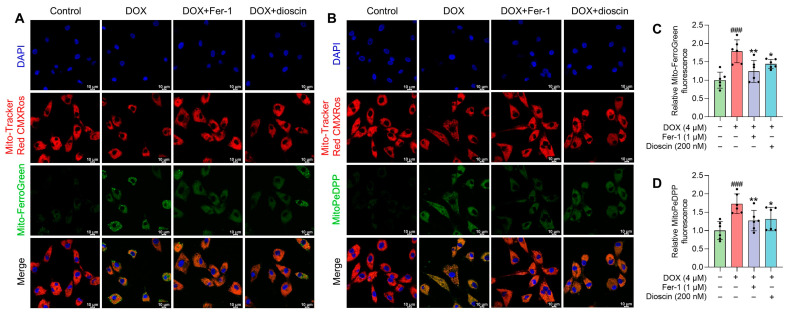
Effects of dioscin on DOX-induced mitochondrial Fe^2+^ and lipid ROS in H9c2 cells. (**A**) Representative Mito-FerroGreen staining images of DOX-induced H9c2 cells treated with or without dioscin and Fer-1. (**B**) The bar chart indicates the relative levels of mitochondrial Fe^2+^ in H9c2 cells. Data are mean ± SD (*n* = 3). (**C**) Representative MitoPeDPP staining images of DOX-induced H9c2 cells treated with or without dioscin and Fer-1. (**D**) The bar chart indicates the relative levels of mitochondrial lipid ROS in H9c2 cells. Data are mean ± SD (*n* = 3). * *p* < 0.05, ** *p* < 0.01 vs. DOX treatment group; ### *p* < 0.001 vs. control group.

Mitochondria, as the primary organelle in DIC, are critical sites for ferroptosis. Electron microscopy revealed significant changes in mitochondrial structure after DOX treatment. To assess the impact on mitochondrial function, changes in mitochondrial membrane potential (MMP) after DOX treatment, with or without dioscin, were evaluated using JC-1 staining. The increase in green fluorescence of JC-1 after DOX treatment indicated MMP disruption (Figure 5A,C). To further analyze the effect of dioscin on mitochondrial ferroptosis, the levels of ROS and Fe^2+^ and the extent of lipid peroxidation in mitochondria were examined. DOX was found to induce significant mitochondrial ROS (mito-ROS) production, but dioscin partially reduced this accumulation in H9c2 cells (Figure 5B,D). Confocal microscopy revealed that the mitochondrial Fe^2+^-specific fluorescence indicator Mito-FerroGreen signal increased following DOX stimulation, while dioscin reduced the accumulation of Fe^2+^ in mitochondria (Figure 6A,C). Additionally, mitochondrial lipid peroxidation, as indicated by MitoPeDPP labeling, increased in the DOX group, but was reduced by dioscin (Figure 6B,D). These results demonstrate that dioscin effectively attenuates changes in mitochondrial ferroptosis indexes, providing strong evidence that dioscin inhibits DOX-induced ferroptosis in cardiomyocytes.

### 3.5. Dioscin Affects Ferroptosis-Related RNA Expression Profiles in DOX-Induced H9c2 Cells

**Figure 7:**
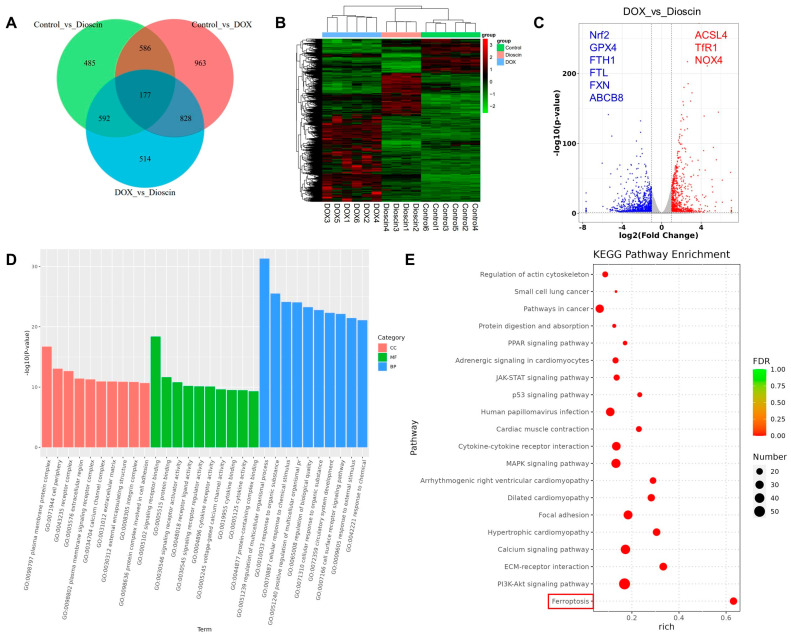
RNA-seq analyses of differentially expressed transcripts. (**A**) Venn diagram of all DEGs identified by RNA sequencing. (**B**) Heatmap of all DEGs identified by RNA sequencing. (**C**) Volcano plot of DEGs between control, DOX, and dioscin groups. (**D**) Gene ontology analysis of differentially expressed genes. (**E**) KEGG analysis of representative signaling pathways.

Transcriptome sequencing was performed to explore the potential molecular mechanisms underlying dioscin’s inhibition of DIC. Notable differences of the expression of 1884 genes were observed between the DOX and control groups, while 2111 genes exhibited significant expression changes between the dioscin and DOX groups (Figure 7A–C). Gene ontology (GO) enrichment analysis revealed that the primary functions affected included plasma membrane protein complex, receptor complex, signaling receptor binding, and regulation of multicellular organismal process. This suggests that dioscin may counteract DIC by modulating various biological activities of H9c2 cells (Figure 7D). KEGG pathway analysis indicated that dioscin could influence pathways related to ferroptosis, PI3K-Akt, p53, JAK-STAT, and PPAR, all of which are associated with myocardial injury (Figure 7E). These pathways are potentially linked to cardiotoxic injuries such as hypertrophic cardiomyopathy, arrhythmogenic right ventricular cardiomyopathy, and dilated cardiomyopathy. Notably, significant differences were found in the expression of genes such as Nrf2, transferrin receptor 1 (TfR1), FTH1, FTL, GPX4, and ACSL4 within the ferroptosis pathway. Taken together, dioscin may exert its protective role against DIC by regulating these targets and associated cardiac ferroptosis-related signaling pathways.

### 3.6. Dioscin Suppresses Cardiac Ferroptosis by Regulating the Nrf2/GPX4 Signaling Pathway in DOX-Induced Rats

**Figure 8:**
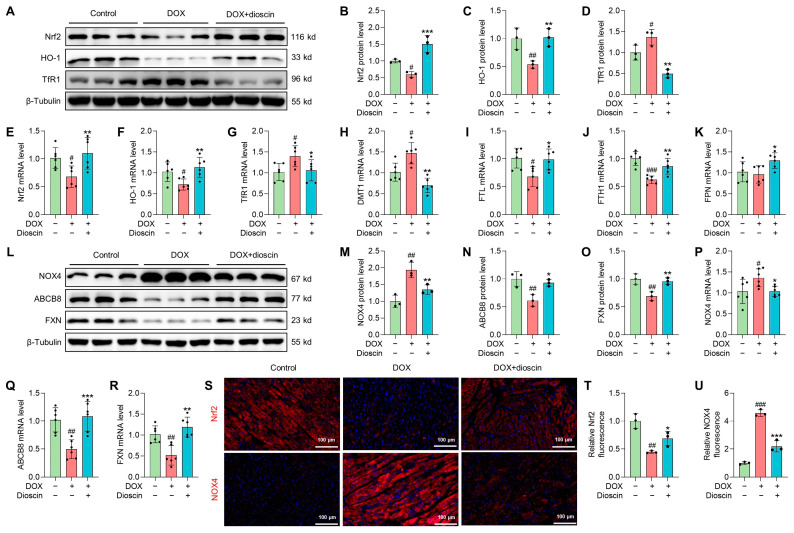
Dioscin suppresses cardiac ferroptosis by regulating the Nrf2/GPX4 signaling pathway in DOX-induced rats. (**A**) Representative Western blotting images of Nrf2, HO-1, and TfR1 in DOX-induced rat treated with or without dioscin. (**B**–**D**) Bar charts indicate the protein expression levels of Nrf2, HO-1, and TfR1 in cardiac tissues. Data are mean ± SD (*n* = 3). (**E**–**K**) Bar charts indicate the mRNA expression levels of Nrf2, HO-1, TfR1, DMT1, FTL, FTH1, and FPN in cardiac tissues. Data are mean ± SD (*n* = 6). (**L**) Representative Western blotting images of NOX4, ABCB8, and FXN in DOX-induced rat treated with or without dioscin. (**M**–**O**) Bar charts indicate the protein expression levels of NOX4, ABCB8, and FXN in cardiac tissues. Data are mean ± SD (*n* = 3). (**P**–**R**) Bar charts indicate the mRNA expression levels of NOX4, ABCB8, and FXN in cardiac tissues. Data are mean ± SD (*n* = 6). (**S**) Representative images of immunofluorescence analysis of Nrf2 and NOX4 expression were captured by fluorescence microscopy. (**T**,**U**) The bar chart indicates the Nrf2 and NOX4 intensity in cardiac tissue. Data are mean ± SD (*n* = 3). * *p* < 0.05, ** *p* < 0.01, *** *p* < 0.001 vs. DOX treatment group; # *p* < 0.05, ## *p* < 0.01, ### *p* < 0.001 vs. control group. (**A**,**L**) Original western blotting figures can be found in Figures S5 and S6.

The regulation of Nrf2-related signaling pathways is considered a potential strategy for the treatment of DIC [27]. Western blot analysis revealed that the expression of Nrf2 and its downstream gene heme oxygenase-1 (HO-1) was decreased in the DOX group. This downregulation was counteracted in the dioscin-treated group (Figure 8A–C,E,F). TfR1, DMT1, FTH1, FTL, and FPN, which have been implicated in ferroptosis by regulating intracellular iron accumulation, are also key downstream targets of Nrf2 [11]. Protein and mRNA expression analyses indicated that dioscin could regulate these genes to inhibit ferroptosis (Figure 8A,D,G–K). NOX4, a crucial enzyme for ROS production in mitochondria, is directly upregulated by DIC, leading to excessive mito-ROS production. In addition, DOX-treated cardiac tissue exhibited a significant reduction in FXN, an essential regulator of mitochondrial iron homeostasis and function. Dioscin’s mitochondrial protection reversed the trend of NOX4 and FXN expression and upregulated mitochondrial iron-output protein ABCB8 (Figure 8L–R). Immunofluorescence analysis further demonstrated that dioscin enhanced Nrf2 expression and inhibited NOX4 expression in myocardial tissue (Figure 8S–U). Activation of the Nrf2/GPX4 pathway by dioscin can further scavenge lipid peroxides produced by mitochondrial damage and thereby limit DOX-induced cardiac ferroptosis.

### 3.7. Dioscin Suppresses Ferroptosis by Regulating the Nrf2/GPX4 Signaling Pathway in DOX-Induced H9c2 Cells

**Figure 9:**
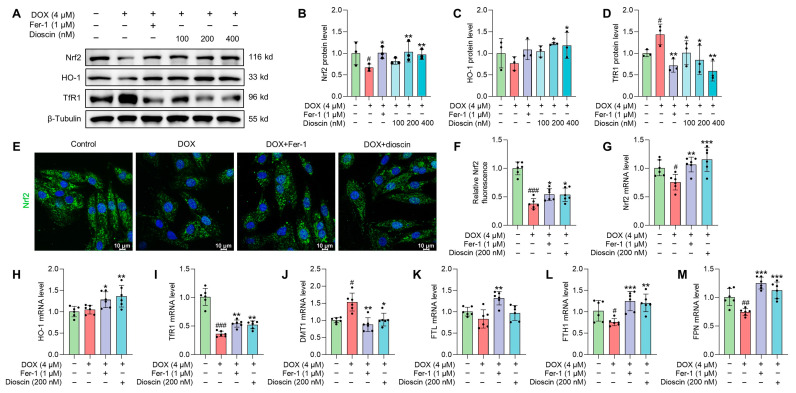
Dioscin suppresses ferroptosis by regulating the Nrf2/GPX4 signaling pathway in DOX-induced H9c2 cells. (**A**) Representative Western blotting images of Nrf2, HO-1, and TfR1 in DOX-induced H9c2 cells treated with or without dioscin and Fer-1. (**B**–**D**) Bar charts indicate the protein expression levels of Nrf2, HO-1, and TfR1 in H9c2 cells. Data are mean ± SD (*n* = 3). (**E**) Representative images of immunofluorescence analysis of Nrf2 expression were captured by fluorescence microscopy. (**F**) The bar chart indicates the Nrf2 intensity in H9c2 cells. Data are mean ± SD (*n* = 3). (**G**–**M**) Bar charts indicate the mRNA expression levels of Nrf2, HO-1, TfR1, DMT1, FTL, FTH1, and FPN in DOX-induced H9c2 cells treated with or without dioscin and Fer-1. Data are mean ± SD (*n* = 6). * *p* < 0.05, ** *p* < 0.01, *** *p* < 0.001 vs. DOX treatment group; # *p* < 0.05, ## *p* < 0.01, ### *p* < 0.001 vs. control group. (**A**) Original western blotting figures can be found in Figure S7.

**Figure 10:**
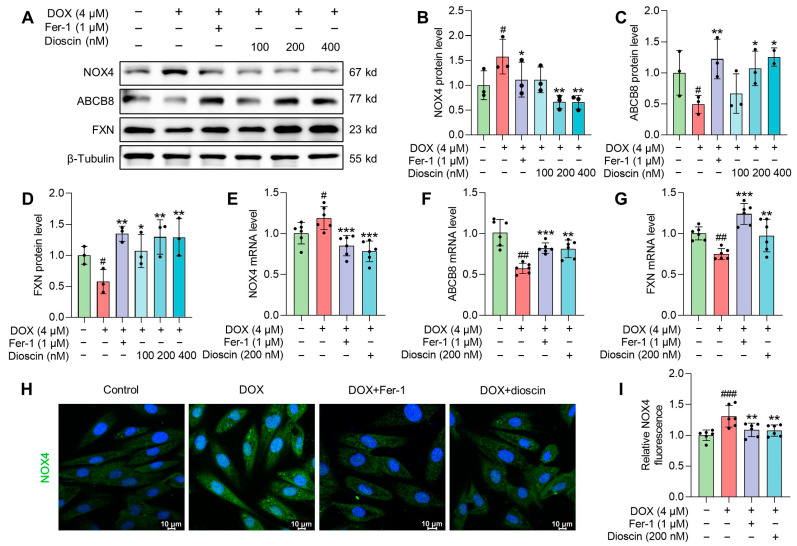
Mechanism of dioscin suppressing DOX-induced mitochondrial damage in H9c2 cells. (**A**) Representative Western blotting images of NOX4, ABCB8, and FXN in DOX-induced H9c2 cells treated with or without dioscin and Fer-1. (**B**–**D**) Bar charts indicate the protein expression levels of NOX4, ABCB8, and FXN in H9c2 cells. Data are mean ± SD (*n* = 3). (**E**–**G**) Bar charts indicate the mRNA expression levels of NOX4, ABCB8, and FXN in DOX-induced H9c2 cells treated with or without dioscin and Fer-1. Data are mean ± SD (*n* = 6). (**H**) Representative images of immunofluorescence analysis of NOX4 expression were captured by fluorescence microscopy. (**I**) The bar chart indicates the NOX4 intensity in H9c2 cells. Data are mean ± SD (*n* = 3). * *p* < 0.05, ** *p* < 0.01, *** *p* < 0.001 vs. DOX treatment group; # *p* < 0.05, ## *p* < 0.01, ### *p* < 0.001 vs. control group. (**A**) Original western blotting figures can be found in Figure S8.

In vitro studies showed that DOX suppressed the expression of Nrf2 and its downstream gene HO-1, whereas dioscin administration reversed this suppression (Figure 9A–C,G,H). Intracellular immunofluorescence in H9c2 cells revealed that dioscin promoted the nuclear translocation of Nrf2 (Figure 9E,F). Furthermore, protein and mRNA analyses indicated that dioscin regulates the downstream iron metabolic targets of Nrf2, including TfR1, DMT1, FTH1, FTL, and FPN, thereby inhibiting intracellular ferroptosis in H9c2 cells (Figure 9A,D,I–M). Meanwhile, dioscin inhibited NOX4 expression and increased FXN and ABCB8 expression to alleviate mito-ROS overproduction and mitochondrial Fe^2+^ accumulation (Figure 10A–G), which was also demonstrated by immunofluorescence (Figure 10H,I). Notably, activation of the Nrf2/GPX4 pathway by dioscin further scavenged lipid peroxides produced by mito-ROS and high iron levels, thereby reversing DOX-induced ferroptosis.

### 3.8. The Influence of Dioscin on Ferroptosis Was Abolished by Nrf2 Inhibition

**Figure 11:**
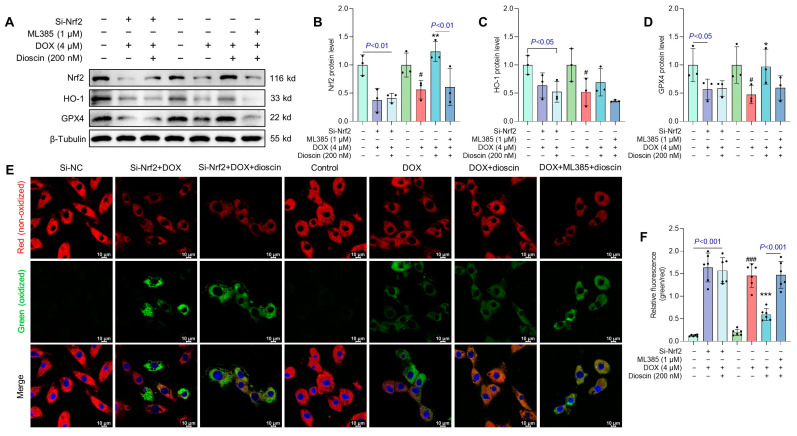
The influence of dioscin on ferroptosis was abolished by Nrf2 inhibition. (**A**) Representative Western blotting images of Nrf2, HO-1, and GPX4 after treatment with Nrf2 siRNA or the Nrf2 inhibitor ML385. (**B**–**D**) Bar charts indicate the protein expression levels of Nrf2, HO-1, and GPX4 after Nrf2 inhibition. Data are mean ± SD (*n* = 3). (**E**) Representative C11 BODIPY 581/591 staining images after treatment with Nrf2 siRNA or the Nrf2 inhibitor ML385. (**F**) The bar chart indicates the relative levels of lipid ROS after Nrf2 inhibition. Data are mean ± SD (*n* = 3). * *p* < 0.05, ** *p* < 0.01, *** *p* < 0.001 vs. DOX treatment group; # *p* < 0.05, ### *p* < 0.001 vs. control group. (**A**) Original western blotting figures can be found in Figure S9.

To further evaluate the protective effect of dioscin against DOX-induced ferroptosis and its dependence on Nrf2 activation, ML385 and specific siRNA knockout were used. Western blot analysis demonstrated that transfection with Nrf2 siRNA and ML385 treatment significantly decreased Nrf2 expression in H9c2 cells (Figure S1E–H). Both ML385 and siRNA reduced the expression of Nrf2, HO-1, and GPX4 (Figure 11A–D), and increased lipid ROS levels (Figure 11E,F). These effects were not attenuated by dioscin treatment. Thus, the inhibition of Nrf2 negated the therapeutic effect of dioscin against ferroptosis.

## 4. Discussion

Despite the lack of specific treatments for DIC, increasing evidence underscores the significance of ferroptosis in DIC [8]. In the present study, we found that the natural steroid dioscin ameliorated DOX-induced cumulative cardiomyopathy in terms of lowering myocardial enzyme levels, ameliorating myocardial pathological injury, and restoring cardiac function. Our results are in agreement with previous reports that showed that DOX exacerbates iron accumulation and lipid peroxidation in cardiac tissue, contributing to cardiotoxicity [15]. Dioscin not only significantly suppressed iron levels in cardiac tissues, but also limited the deleterious accumulation of ROS and lipid peroxides MDA and 4-HNE, which are strongly associated with ferroptosis. ACSL4 esterifies CoA into specific polyunsaturated fatty acids and undergoes lipid peroxidation (PL-PUFA-OOH) in the presence of O_2_ and Fe^2+^ to enhance sensitivity to ferroptosis activation [28]. Dioscin mitigated DOX-induced abnormal expression of ACSL4. Meanwhile, dioscin and Fer-1 decreased the aggregation of Fe^2+^ and lipid peroxides in DOX/erastin-stimulated H9c2 cells. Importantly, dioscin could ameliorate the ferroptosis-generated characteristic mitochondrial structural changes. These results suggest that dioscin’s cardioprotective effects are closely linked to its ability to inhibit ferroptosis.

Transcriptome analysis revealed that dioscin significantly influences the ferroptosis signaling pathway, particularly impacting key targets such as Nrf2, TfR1, FTH, FTL, GPX4, and ACSL4. Emerging evidence indicates that Nrf2 not only regulates the antioxidant system associated with DOX-induced ferroptosis, but also regulates iron storage and delivery [29]. Firstly, our study revealed that dioscin activates Nrf2 and its downstream HO-1 to enhance antioxidant enzyme activities, thereby inhibiting DOX-induced oxidative stress. Iron is physiologically absorbed by TfR1 (Fe^3+^) or DMT1 (Fe^2+^) and temporarily stored in ferritin (FTH1/FTL). Excess iron is expelled by FPN to maintain intracellular iron homeostasis [30]. However, DOX-induced dysregulation of these iron metabolism genes contributes to increased iron uptake and storage, exacerbating susceptibility to lipid peroxidation [11,12]. Under conditions of Nrf2 activation, dioscin inhibited the expression of TfR1 and DMT1 and promoted iron storage and elimination through upregulating FTH1/FTL and FPN expression, thereby inhibiting intracellular iron accumulation. Importantly, dioscin regulates these iron metabolism genes independently of hepcidin (Figure S1I,J). GPX4, a crucial gene in defense against ferroptosis, functions in both the cytoplasm and mitochondria. Its activity depends on GSH, which is produced via the cystine/solute carrier family 7 member 11 (SLC7A11) axis [31]. Studies have shown that Nrf2 directly affects GSH synthesis and promotes GPX4 expression to reduce toxic PL-PUFA-OOH to non-toxic PL-PUFA-OH and water, thus inhibiting ferroptosis [32,33]. DIC has been proven to downregulate Nrf2/GPX4-driven antioxidant capacity and promote lipid peroxidation production in cells or mitochondria [14,15]. Dioscin treatment could activate the Nrf2/GPX4 pathway, promote Nrf2 expression and nuclear transmutation, reduce GSH and GPX4 consumption, regulate intracellular iron metabolism balance, and clear lipid peroxides induced by high iron. This mechanism is crucial in preventing DOX-induced ferroptosis, as it directly combats the lipid peroxidation that is central to ferroptotic cell death.

The high affinity of DOX for mitochondria leads to excessive mito-ROS production and iron accumulation, leading to lipid peroxide formation and mitochondrial damage [34]. NOX4 is the major oxidase for ROS production in cardiac mitochondria, and its expression is significantly elevated in the DIC model [35]. High NOX4 expression is positively correlated with ferroptosis [36]. In this study, dioscin inhibited DOX-promoted NOX4 expression and mitochondrial ROS levels. FXN is a central target of mitochondrial iron metabolism because of its iron-binding properties and its involvement in iron-sulfur cluster (ISC) and heme synthesis [37]. Previous studies have shown that DOX blocks the stability of FXN, leading to mitochondrial iron accumulation and subsequent ROS formation [38]. ABCB8 is an ABC transporter protein located in the inner mitochondrial membrane that regulates mitochondrial iron export and DOX retention in mitochondria [39]. Our data indicate that dioscin upregulates FXN and ABCB8 expression to reduce DOX-induced iron accumulation. Nrf2 regulates ROS production through mitochondrial NADPH oxidase and activates mitochondrial iron regulatory protein FXN to prevent mitochondrial dysfunction [12,40,41]. Therefore, activation of the Nrf2/GPX4 pathway further removes lipid peroxides produced by mitochondrial damage, thereby inhibiting the occurrence of ferroptosis. However, Nrf2 silencing and ML385 treatment reversed the beneficial effects of dioscin, including reversal of HO-1 and GPX4 expression as well as increased intracellular lipid peroxidation. After summarizing the results of DOX-induced ferroptosis, we found that dioscin may be a potential ferroptosis inhibitor, similar to Fer-1, in regulating DOX-stimulated ferroptosis labeling and mitochondrial protection (Table S1). Taken together, these results confirm the essential contribution of dioscin in mitigating the Nrf2/GPX4 axis to attenuate ferroptosis and highlight the potential of dioscin as a therapeutic agent against DIC.

## 5. Conclusions

In conclusion, dioscin appears to be a potent inhibitor of DOX-induced ferroptosis by modulating the Nrf2/GPX4 signaling axis. Its multifaceted effects include regulating iron metabolism, clearing lipid peroxides, and maintaining mitochondrial integrity, all of which are critical for mitigating DIC.

## Figures and Tables

**Table 1:** Primer sequences for RT-qPCR.

Gene	Forward Primer (5′-3′)	Reverse Primer (5′-3′)
Nfe2l2 (Nrf2, rat)	GCATTTCGCTGAACACAA	CTCTTCCATTTCCGAGTCA
Hmox1 (HO-1, rat)	AGAGTTTCTTCGCCAGAGG	GAGTGTGAGGACCCATCG
NOX4 (rat)	CCTCAGTCAAACAGATGGG	CCACAGCAGAAAACTCCAA
GPX4 (rat)	GGACCTGCCGTGCTATCT	GGCCTCTGGACCTTCCTC
ACSL4 (rat)	ATCAAGAAGGGAAGCAAGG	ACAAAGAGGGGCATCATATC
Tfrc (TfR1, rat)	TGGAGAACCTGAGACTTCG	TCACCAGAGAGGGCATTT
Slc11a2 (DMT1, rat)	GGTGGTGGAGCCGAATCCTAT	GTGGGAGGGAAGAGTTGCTG
FTH1 (rat)	TGCGCCAGAACTTTCAC	GTACAAGGCCACATCATCC
FTL (rat)	GTCTTCGCGGTTAGCTCCAT	CAACTTGAGGAGACGCTCGG
Slc40a1 (FPN, rat)	CAAAGGACCAAAGACCGA	CCCATAATCTCCGTCAGC
FXN (rat)	AACCTCCACTACCTCGGTCA	CTAGAGAGCTTGGGTTGCCC
ABCB8 (rat)	CAAGTCTGTGAGGCTGGGA	CTGAGGGCAGTGAGGCA
Hepcidin (rat)	GATGGCACTAAGCACTCGGA	GCAGAGCCGTAGTCTGTCTC
GAPDH (rat)	GAAGGTCGGTGTGAACGGAT	CCCATTTGATGTTAGCGGGAT

## Data Availability

The data are available on reasonable request from the corresponding author.

## Supplementary Materials

The following supporting information can be downloaded at: https://www.mdpi.com/article/10.3390/biom14040422/s1, Figure S1: Results of cell viability, Nrf2 inhibition, and hepcidin mRNA expression; Figure S2: Effects of dioscin on Nrf2, HO-1, GPX4, ACSL4, and ABCB8 protein expression in H9c2 cells; Figure S3: Original western blotting figures for Figure 2K; Figure S4: Original western blotting figures for Figure 3M; Figure S5: Original western blotting figures for Figure 8A; Figure S6: Original western blotting figures Figure 8L; Figure S7: Original western blotting figures for Figure 9A; Figure S8: Original western blotting figures for Figure 10A; Figure S9: Original western blotting figures for Figure 11A; Table S1: Summary of Fer-1 and dioscin inhibition of DOX-induced ferroptosis.

## Abbreviations

4-HNE, 4-hydroxynonenal; ABCB8, ATP-binding cassette B8; ACSL4, acyl-CoA synthetase long-chain family 4; CAT, catalase; CK-MB, creatine kinase isoenzyme-MB; ALT, alanine transaminase; CO, cardiac output; AST, aspartate aminotransferase; cTnT, cardiac troponin T; DMT1, divalent metal transporter; DIC, doxorubicin-induced cardiotoxicity; FPN, ferroportin; GPX4, glutathione peroxidase 4; Fer-1, ferrostatin-1; FTH1, ferritin heavy chain; FXN, frataxin; GSH, glutathione; GSH-Px, glutathione peroxidase; FTL, ferritin light chain; HO-1, heme oxygenase-1; LDH, lactate dehydrogenase; MDA, malondialdehyde; mTOR, mammalian target of rapamycin; NOX4, nicotinamide adenine dinucleotide phosphate oxidase 4; Nrf2, nuclear factor-erythroid 2-related factor 2; LVEF, left ventricular ejection fraction; PDK1, phosphoinositide-dependent protein kinase-1; PUFA, polyunsaturated fatty acids; LVFS, left ventricular fractional shortening; ROS, reactive oxygen species; SLC7A11, solute carrier family 7 member 11; SOD, superoxide dismutase; SV, stroke volume; TfR1, transferrin receptor.
