# Supplementary material for: Protective Role of Dioscin against Doxorubicin-Induced Chronic Cardiotoxicity: Insights from Nrf2-GPX4 Axis-Mediated Cardiac Ferroptosis
Source: Biomolecules. 2024 Mar 30;14(4):422. doi: 10.3390/biom14040422 (PMC11047995; doi:10.3390/biom14040422)
Supplement: Supplementary file 1 [file biomolecules-14-00422-s001.zip › biomolecules-2886715-supplementary.pdf]

Supplementary Materials:

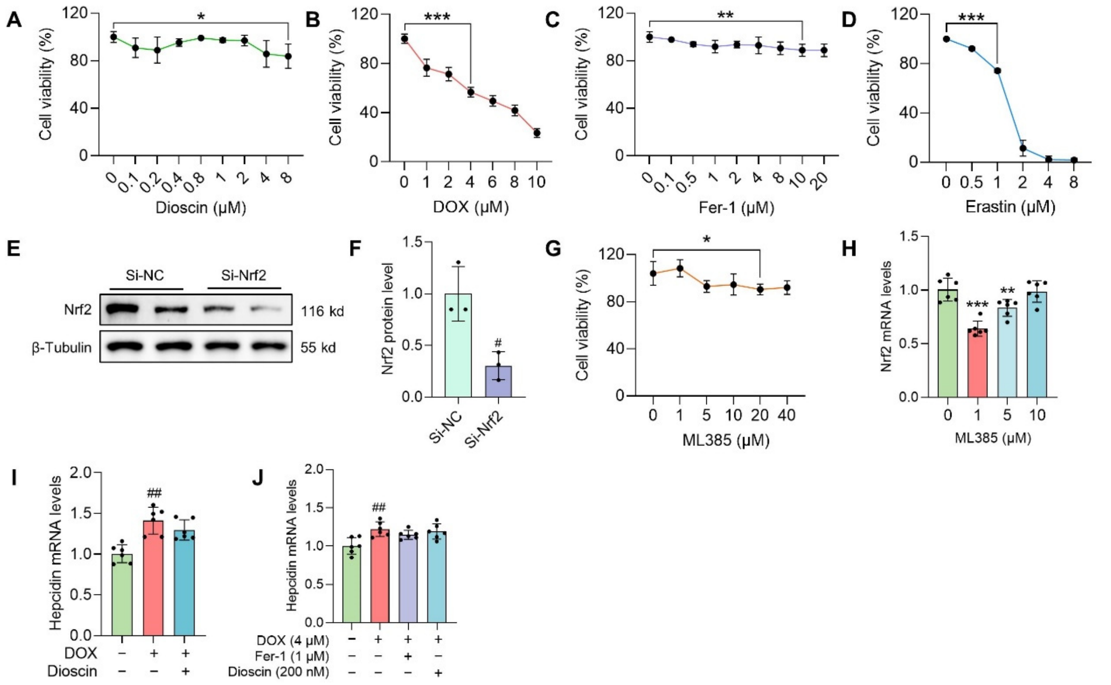

Figure S1. Results of cell viability, Nrf2 inhibition, and hepcidin mRNA expression. (A) The cell viability of H9c2 cells treated with dioscin at indicated concentrations. (B) The cell viability of H9c2 cells treated with DOX at indicated concentrations. Data are mean ± SD (n = 6). (C) The cell viability of H9c2 cells treated with Fer-1 at indicated concentrations. Data are mean ± SD (n = 6). (D) The cell viability of H9c2 cells treated with erastin at indicated concentrations. Data are mean ± SD (n = 6). (E) Representative western blotting images of Nrf2 after transfection with siRNA. (F) Bar charts indicate the protein expression levels of Nrf2 after transfection with siRNA. Data are mean ± SD (n = 3). (G) The cell viability of H9c2 cells treated with ML385 at indicated concentrations. Data are mean ± SD (n = 6). (H) Bar charts indicate the mRNA expression levels of Nrf2 after treatment with ML385 at indicated concentrations. Data are mean ± SD (n = 6). (I and J) Bar charts indicate the mRNA expression levels of hepcidin in cardiac tissues and H9c2 cells. Data are mean ± SD (n = 6). * $p < 0.05$, ** $p < 0.01$, *** $p < 0.001$ vs Control group. # $p < 0.05$ vs Si-NC group.

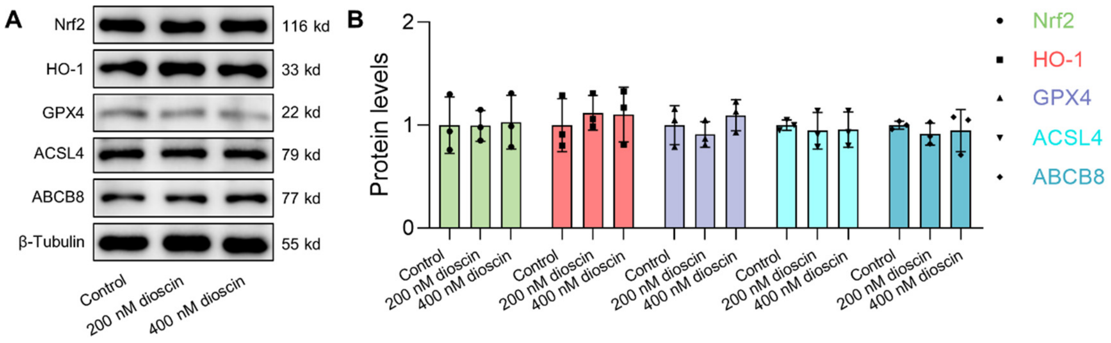

Figure S2. Effects of dioscin on Nrf2, HO-1, GPX4, ACSL4, and ABCB8 protein expression in H9c2 cells. (A) Representative western blotting images of Nrf2, HO-1, GPX4, ACSL4, and ABCB8 in H9c2 cells treated with dioscin (200 and 400 nM). (B) Bar charts indicate the protein expression levels of Nrf2, HO-1, GPX4, ACSL4, and ABCB8 in H9c2 cells. Data are mean ± SD (n = 3).

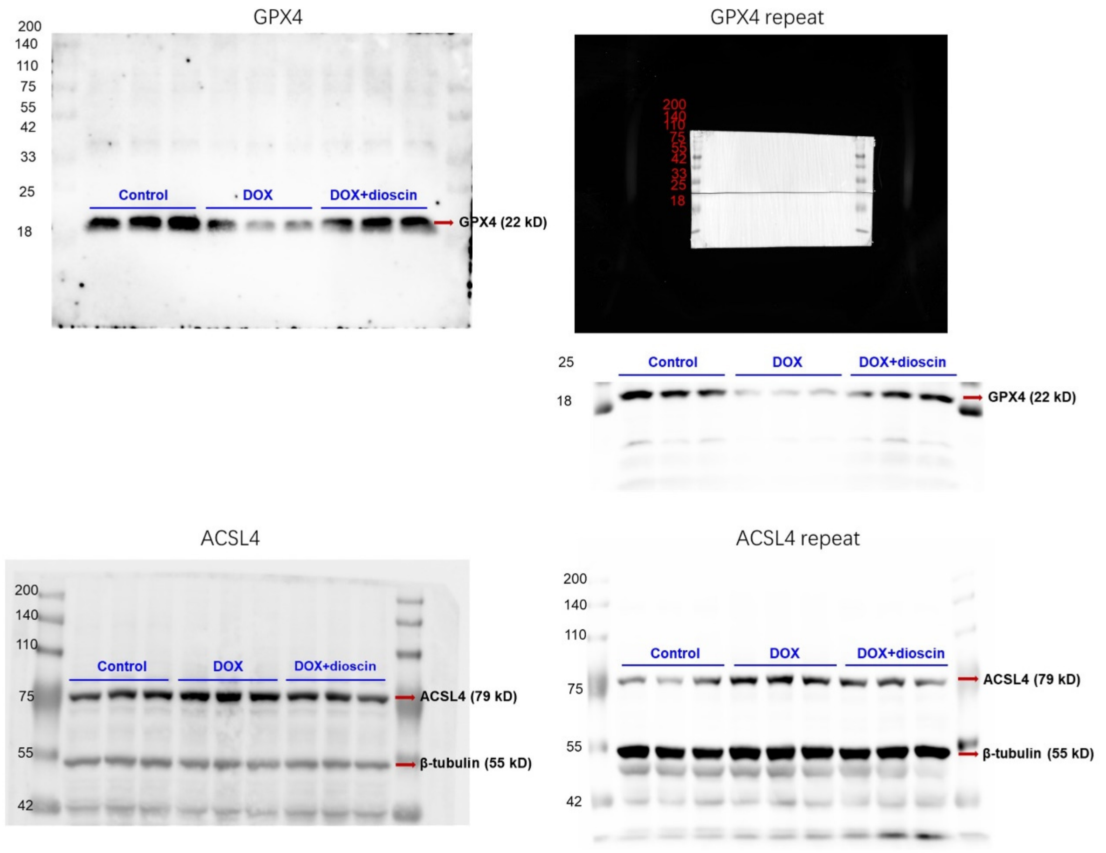

Figure S3. Original western blotting figures for Figure 2K.

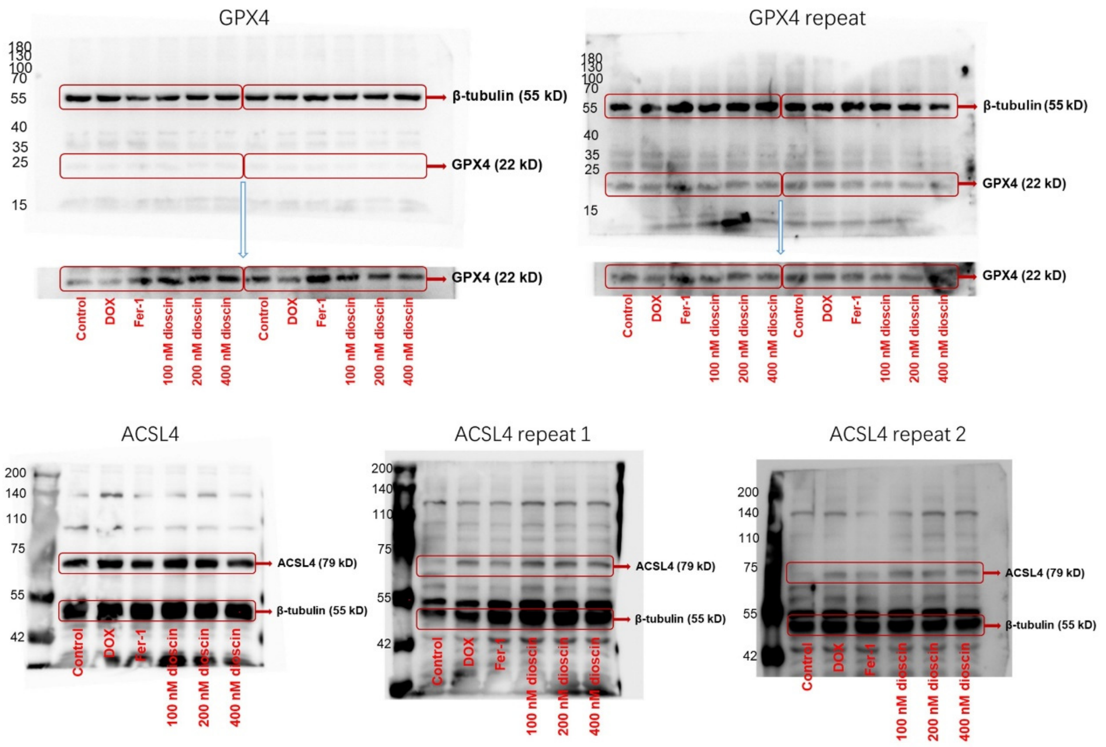

Figure S4. Original western blotting figures for Figure 3M.

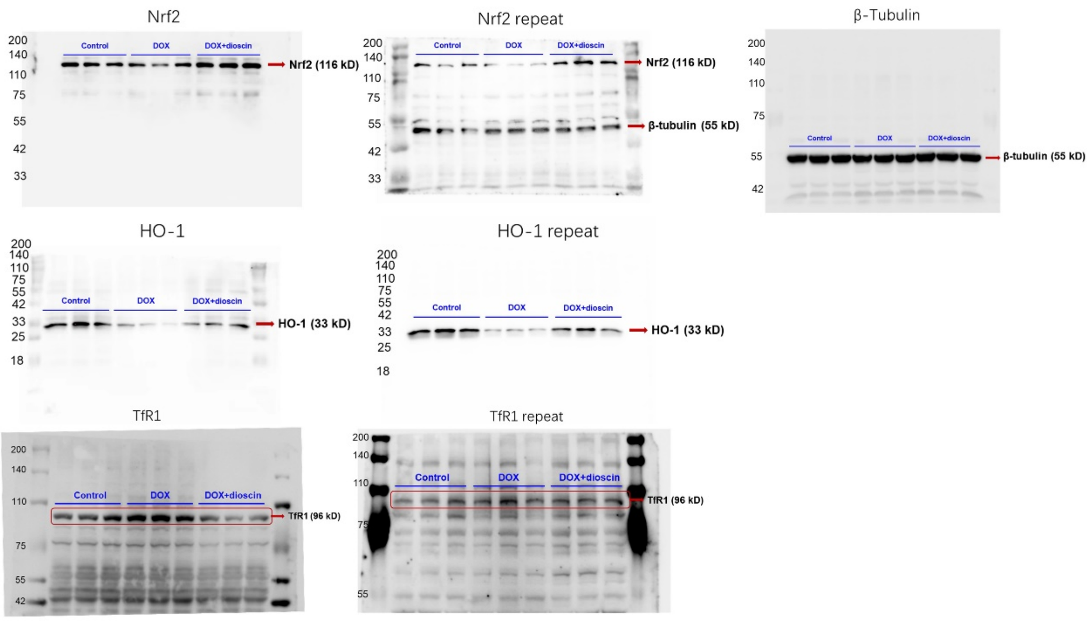

Figure S5. Original western blotting figures for Figure 8A.

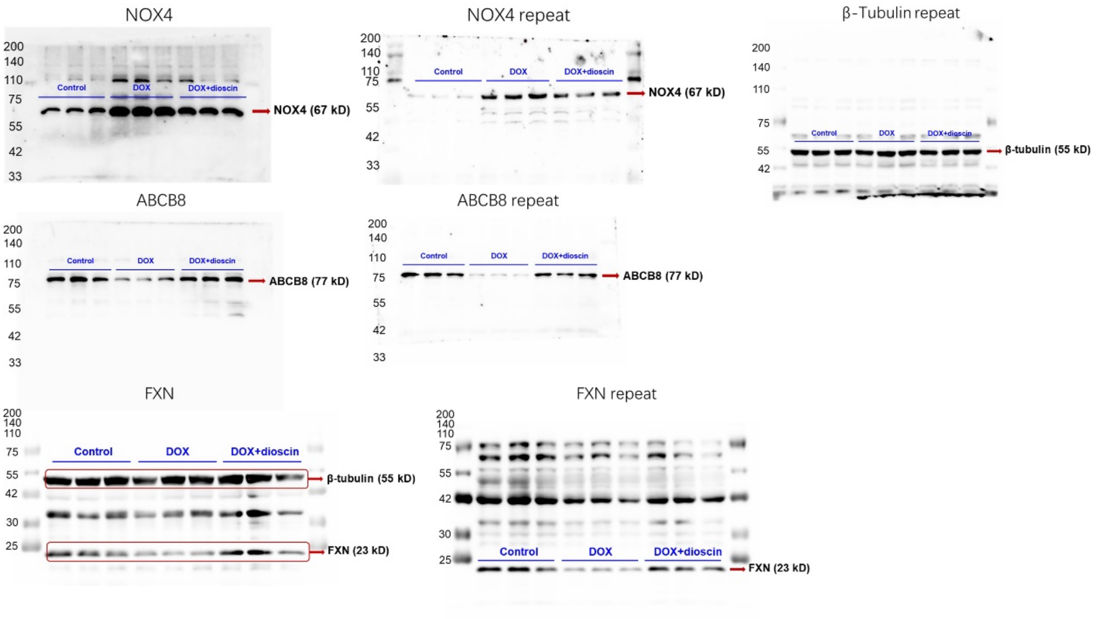

Figure S6. Original western blotting figures Figure 8L.

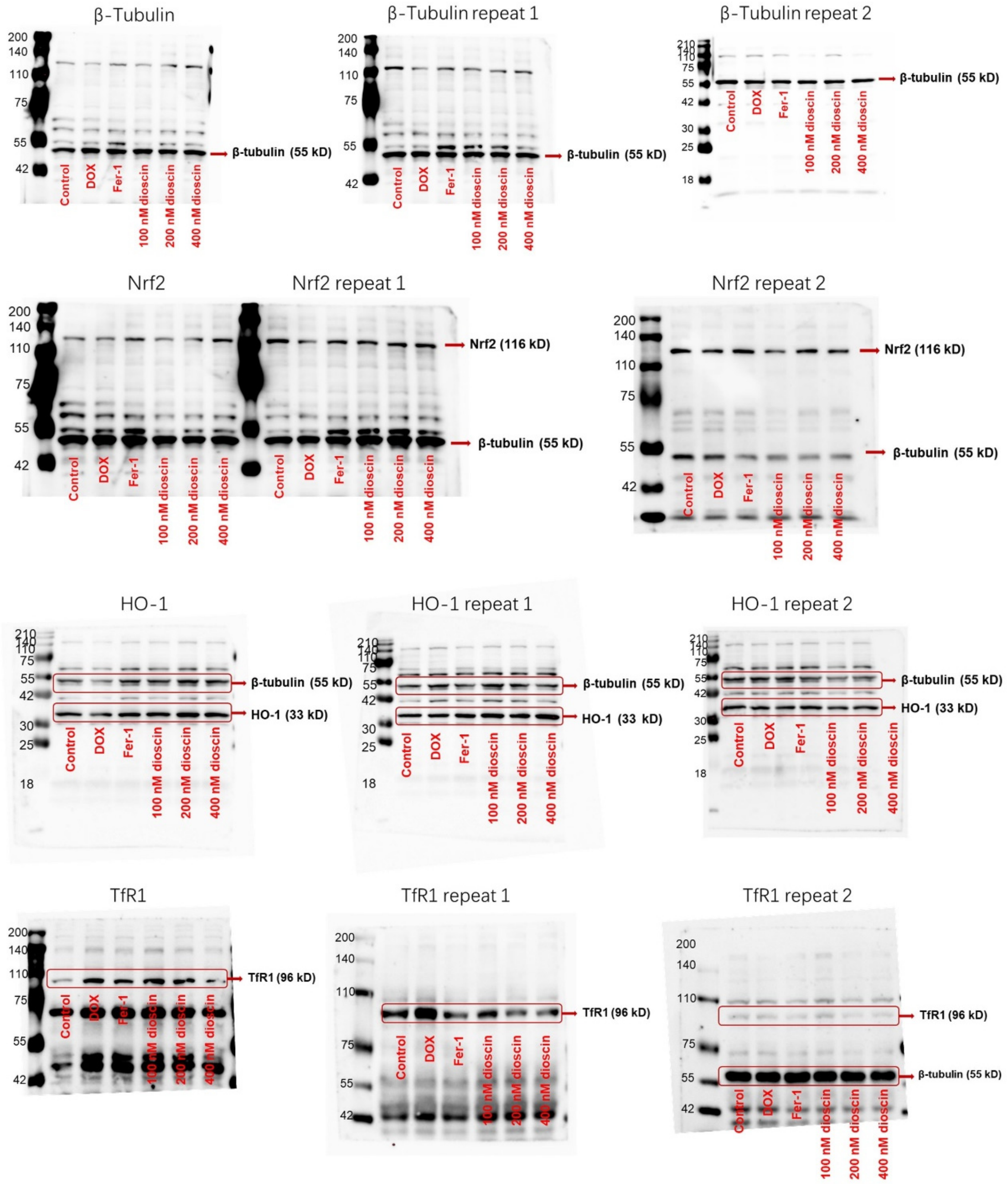

Figure S7. Original western blotting figures for Figure 9A.

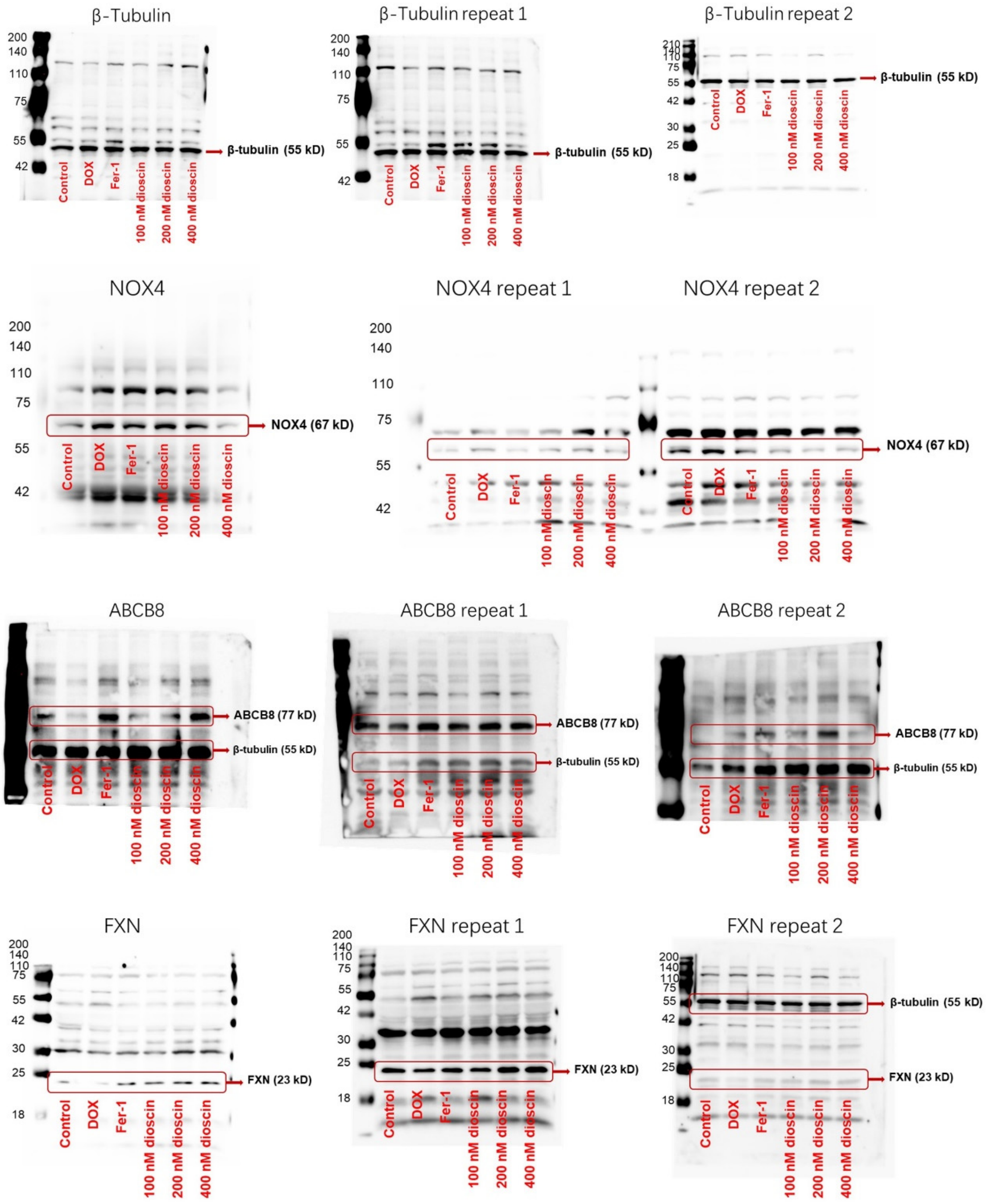

Figure S8. Original western blotting figures for Figure 10A.

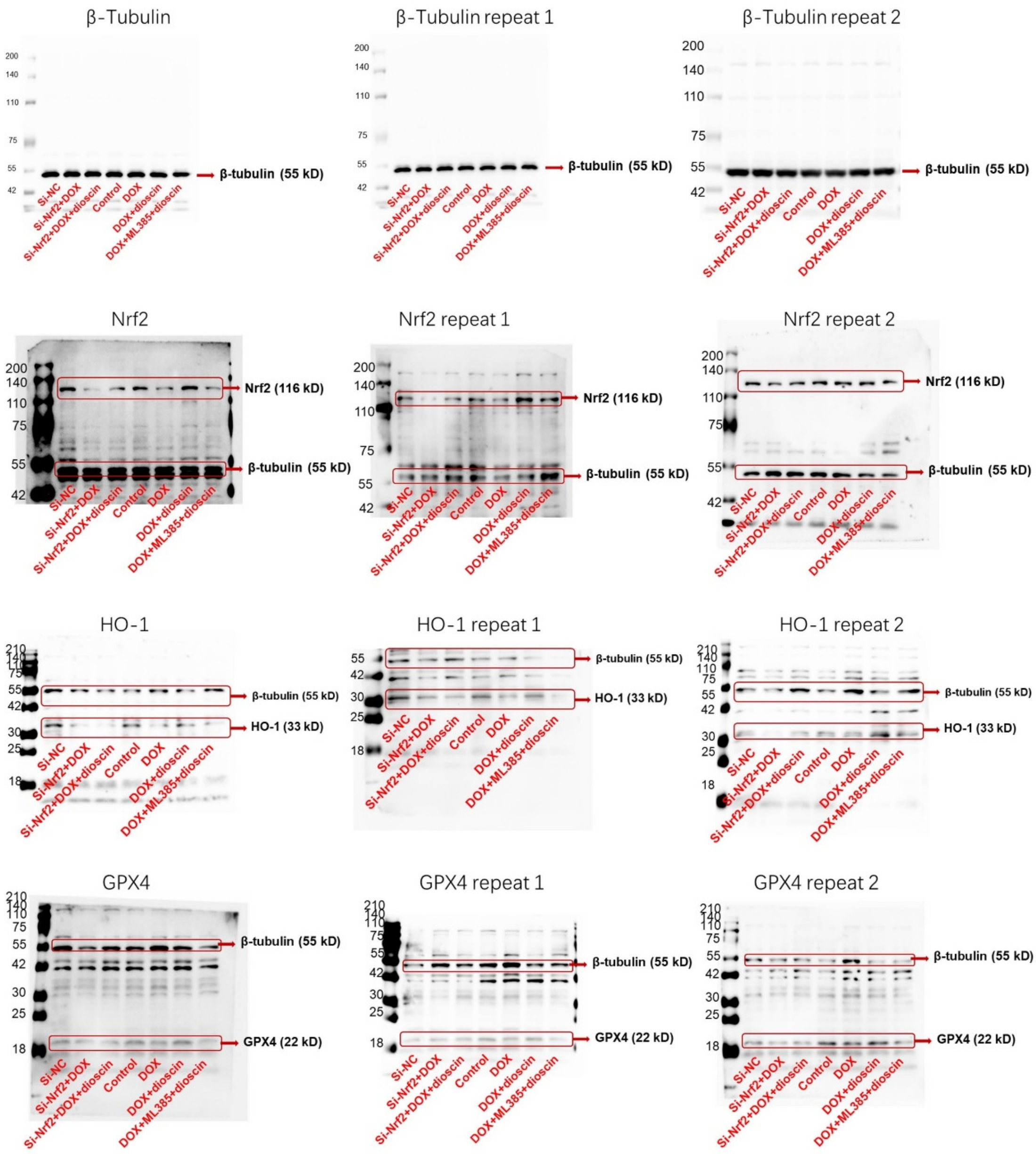

Figure S9. Original western blotting figures for Figure 11A.

Table S1. Summary of Fer-1 and dioscin inhibition of DOX-induced ferroptosis.

DOX	Fer-1	dioscin
(+) ROS levels	(-)	(-)
(-) Antioxidant enzyme activity	(+)	(+)
(+) 4-HNE and MDA levels	(-)	(-)
(+) ACSL4 expression	(-)	(-)
(-) GPX4 expression	(+)	(+)
(+) Intracellular Fe2+ and lipid peroxides	(-)	(-)
(-) Mitochondrial membrane potential	(+)	(+)
(+) Mitochondrial ROS	(-)	(-)
(+) Mitochondrial Fe2+ and lipid peroxides	(-)	(-)
(-) Nrf2 and HO-1 expression	(+)	(+)

(+) TfR1 expression	(-)	(-)
(+) DMT1 and hepcidin expression	(-)	(-)
(-) FTH1, FTL and FPN expression	(+)	(+)

(+) promote or improve (-) repress or reduce.
